# Jefferson Medical College

**Published:** 1892-12

**Authors:** 


### A NEW PROFESSORSHIP IN THE JEFFERSON MEDICAL COLLEGE.

At a meeting of the board of trustees held on Wednesday, November 30th, 1892, Dr. G. E. de Schweinitz was, on the unanimous recommendation of the faculty, elected Clinical Professor on Ophthalmology in the Jefferson Medical College.

At the time of election Dr. de Schweinitz was Professor of Ophthalmology in the Philadelphia Polyclinic and Lecturer on Medical Ophthalmoscopy in the University of Pennsylvania.

---

MENTHOL IN VOMTING OF PREGNANCY.—Dr. Moreiil, Wreizcalling the work of Gottschalk, Weiss, Lahnstein, Drews, Graefe, Kaltenbach, Lomer, Jaffe, Piza, and May, concerning the use of this drug for vomiting, records its successful use in a case under his own observation, in which muriatic acid, bicarbonate of soda, ice, cherry-laurel water, morphine, bromide of soda, hyoscyamus, chloral, and cocaine had failed. The formulæ employed have been those of Gottschalk (menthol, 1; alcohol, 20; distilled water, 150; a dessertspoonful every two or three hours); of Weiss (menthol, 1; alcohol, 20; syrup, 30; which makes a better solution); of the author (10 drops of a 20 per cent. solution in olive oil, dropped in finely-powdered sugar), the last formula leaving only a sweet taste in the mouth, after a little water has been drunk. The dose of menthol for this purpose is about a grain.—*Centralblatt fur die gstammte Therapie*, 1892, Heft. 8, S. 449.

